# Organic–inorganic interactions revealed by Raman spectroscopy during reversible phase transitions in semiconducting [(C_2_H_5_)_4_N]FeCl_4_†

**DOI:** 10.1039/d1ra02475b

**Published:** 2021-05-24

**Authors:** Khaoula Ben Brahim, Malika Ben gzaiel, Abderrazek Oueslati, Kamel Khirouni, Mohamed. Gargouri, Gwenaël Corbel, Jean-François Bardeau

**Affiliations:** Laboratoire de Caractérisation Spectroscopique et Optique des Matériaux, Faculté des Sciences, Université de Sfax B.P. 1171 3000 Sfax Tunisia benbrahimkhawla75@gmail.com; Laboratoire de Physique des Matériaux et des Nanomatériaux appliquée à l'Environnement, Faculté des Sciences de Gabes, Université de Gabes cite Erriadh, 6079 Gabes Tunisia; Institut des Molécules et Matériaux du Mans (IMMM), UMR-6283 CNRS, Le Mans Université Avenue Olivier Messiaen F-72085 Le Mans Cedex 9 France

## Abstract

The alkylammonium halogenoferrate families are subjected to diverse studies according to their wide field application. However, these compounds show various transitions depending on the preparation process. In this paper, the [(C_2_H_5_)_4_N]FeCl_4_ compound was successfully synthesized using a slow evaporation solution growth method at room temperature. An optical absorption measurement confirms the semiconductor nature with a band gap around 2.95 eV. The X-ray powder diffraction (XRPD) data confirmed the formation of a single-phase with hexagonal-type structure. The differential scanning calorimetry (DSC) indicated that the [(C_2_H_5_)_4_N]FeCl_4_ compound undergoes eight reversible phase transitions between 193 and 443 K. At high temperature (*T* > 423 K) the plastic nature of the crystals was confirmed. Temperature-controlled X-ray diffraction reveals that the thermal expansion of the crystal structure is non homothetic in the (*a*,*b*) plane and along the *c* axis. The temperature dependence of the Raman spectra up to 443 K revealed specific reorientations and molecular displacements of the organic and inorganic components associated with the phase transitions. We aim to thermally stabilize the [(C_2_H_5_)_4_N]FeCl_4_ compound which has a band gap suitable for photocatalytic processes.

## Abstract 2

The alkylammonium halogenoferrate families are subjected to diverse studies according to their wide field application.

## Graphical abstract

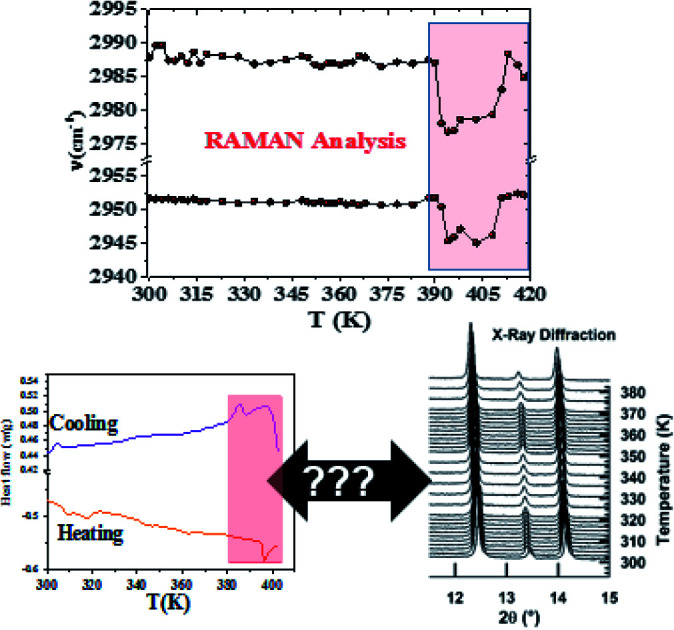

## Introduction

An organic–inorganic hybrid material is a multi-component compound having at least an organic or inorganic group with a characteristic length on the nanometer scale.^1^ Benefiting from the combination of organic cations and inorganic anions, hybrid materials may demonstrate better properties compared to their individual counterparts. Indeed, by adjusting the chemical composition, it is possible to modify the interactions at the molecular level and generate materials with improved performance or create multifunctional materials with interesting physicochemical properties.^2,3^

Plastic crystals, described in the 1960's,^4^ represent an emerging family of hybrid materials, due to very specific multifunctional properties such as higher ionic conductivity and reversible changes in their dielectric, conducting and magnetic behavior.^5–9^

The plastic phase is typically reached by one or more solid–solid phase transitions on warming the fully ordered crystalline phase. It can be considered as a mesophase between a crystalline and a liquid like state which acquires some rotational degree of freedom above a certain temperature. This state is characterized by a long-range order but short-range disorder, which typically originates from rotational motions of the molecules while their center of gravity remains fixed in the crystal lattice.^10–12^ So, the plastic phase transition is between a high-symmetric (usually cubic) and a low symmetric crystal phase which usually accompanies with much larger enthalpy change.^6^ Recently, typical examples which combine organic cations and inorganic chloro-ferrate anions have been reported. High ionic conductivity in plastic choline [FeCl_4_] at room temperature makes it of interest as solid state ionic conductors.^10^ The [(CH_3_)_3_S][FeCl_4_] is a very interesting plastic hybrid compound with coexistence of multifunctional properties that can be useful for both solar thermal and electric energy storage.^11^

Perovskite hybrid crystalline materials have rich optoelectronic applications such as in solar cell light absorbers, light emitting diodes, light detectors due to their tunable bandgaps and high carrier mobility.^13^ Compared to conventional organic–inorganic materials, phase transition hybrid compounds have gained increasing attention due to their potential applications in communication, data storage, sensing, switchable dielectric devices.^14^ In general, organic cations provide structural flexibility and dynamic behavior (disordered at high temperature and order at low temperature) while inorganic anions, as frameworks, provide stability. A number of complexes with a tetrahedral tetrachloroferrate(iii) anion [FeCl_4_]^−^ mixed with organic cation have been reported for their interesting catalytic properties.^15–18^ For instance, the tetraethylammonium tetrachloroferrate ([(C_2_H_5_)_4_N]FeCl_4_) compound has already been used as photocatalyst for the oxidation of the cyclohexane^19^ and the toluene^20^ under visible light. This is a notable advantage over TiO_2_ compound which requires ultraviolet (UV) irradiation to induce a photocatalytic oxidation of toluene.^21^ Nevertheless, despite the importance of tetraethylammonium tetrachloroferrate, the phase transitions at high temperature (*T* > 273 K) remain misunderstood. Recent technological applications require a higher operating temperature, such as the battery of an auto-switching cell phone. So, it is therefore crucial to better explore the physical properties of switchable materials and investigate, with accuracy, their reversible phase transitions at high temperatures (*T* > 273 K).^22^

For the quaternary amine cations [(C_*n*_H_2*n*+1_)_4_N]^+^ bound to an iron(iii) chloride, the increase in the number of carbon atoms in the alkyl chain makes it possible to decline the structural stability of the anion (FeCl_4_).^23^ The transition temperatures are dependent on the thermal history of the samples^24^ and contrary results are reported in literature^23,25–27^ for the [(C_2_H_5_)_4_N]FeCl_4_ (*n* = 2) compound. Indeed, the Differential Thermal Analysis (DTA) measurements performed by D. Wyrzykowski *et al.* (2008)^23^ show only one structural phase transition at 427 K related to a change in the spatial arrangement of the hydrocarbon chain of [(C_2_H_5_)_4_N]^+^ cations. A Differential Scanning Calorimetry (DSC) and vibrational studies performed by Ben Brahim *et al.*^25^ as a function of temperature reveal the presence of two phase transitions at 413 and 430 K, respectively. Below the ambient temperature, the changes in the molar heat capacity *C*_pm_, of [(C_2_H_5_)_4_N]FeCl_4_, attest of the presence of three structural phase transitions at ≈235, ≈227 and ≈217 K, respectively.^26^ Although the phase transition at ≈217 K was not detected by X-ray diffraction on single crystal,^27^ the three phase transitions are likely due to the reorientations of ethyl groups.

In the present study, experimental UV-vis spectrum absorption was performed in order to discuss the electronic transitions within the [(C_2_H_5_)_4_N]FeCl_4_ compound. A combination of differential scanning calorimetry (DSC), X-ray powder diffraction (XRPD) and Raman spectroscopy were used to investigate the structural phase transitions of [(C_2_H_5_)_4_N]FeCl_4_ above room temperature.

## Experimental details

The [(C_2_H_5_)_4_N]FeCl_4_ crystals were grown by a slow evaporation at room temperature of a mixed aqueous solution of FeCl_3_ (Purity 98%; FLUKA) and [(C_2_H_5_)_4_N]Cl (Purity 97%; FLUKA). Details of the crystal growth procedure were described elsewhere.^25^

The phase purity of the as-prepared powder was first checked by recording X-ray powder diffraction (XRPD) pattern at room temperature on a PANalytical *θ*/*θ* Bragg–Brentano Empyrean diffractometer (Cu Kα_1+2_ radiations) equipped with the PIXcel^1D^ detector. XRPD pattern was collected at room temperature in the [5°–100°] scattering angle range, with a 0.0131° step size, for a total acquisition time of 10 h.

The absorption and reflectance spectroscopy were performed on powder at room temperature using a Shimadzu UV-3101PC scanning spectrophotometer with a wavelength radiation varying from 200 to 800 nm. The device allows the measure of the absorbance and the reflectance by the external mode using an integrating sphere and a xenon lamp. The barium sulfate powder (BaSO_4_) is used to record the reference signal.

Thermal stability in air of the as-prepared powder was studied by temperature-controlled X-ray powder diffraction. Prior to data collection, a dehydration of the sample was carried out at 373 K for 110 min in the reactor chamber. Then, temperature-controlled XRPD patterns were recorded on the same diffractometer between 298 and 383 K (heating rate of 10 K min^−1^, temperature stabilisation for 20 min, cooling rate of 60 K min^−1^, air flow of 40 mL min^−1^) by using an XRK 900 Anton Paar reactor chamber. The sample was deposited on the sieve (pore size *Φ* 0.2 mm) of the open sample holder cup, both made of glass ceramic Macor®, thus allowing air to flow through the sample. For each temperature, the XRPD pattern was collected in the [10°–50°] scattering angle range, with a 0.0131° step size, for a total acquisition time of 90 min. The refinements of the XRPD pattern were carried out by the Le Bail method^28^ of the FullProf program.^29^

Differential scanning calorimetry (DSC) measurements were performed using a Perkin Elmer DSC-7 instrument at heating/cooling rate of 5 K min^−1^. Two successive heating and cooling runs were carried out in the temperature range from 193 K to 443 K in order to determine the exact phase transitions temperatures. A second series of DSC analysis was carried out between 193 and 406 K to avoid passing through plastic state which allows to disturb the system. The enthalpy changes, (Δ*H*), due to the observed endothermic event were calculated by numerical integration of a surface area under the curve of the heat flow as a function of time.

The Raman spectra were collected between 70 and 3100 cm^−1^ over the temperature range 294–443 K using a T-64000 (Jobin-Yvon, Horiba group, Kyoto, Japan) spectrometer. Prior to data collection, the sample was heated up to 373 K for 10 min then cooled to room temperature. We used a BX41 Olympus microscope equipped with a MSPlan 50x (N.A. 0.55) objective to focus the incident wavelength radiation at 647.1 nm, provided by an argon–krypton ion laser (Innova, Coherent, France). During the experiment, the laser power was less than 2 mW on the sample and the Raman spectra were systematically recorded twice with an integration time of 60 s. The scattered light was then analyzed by a spectrometer with a single monochromator (600 gratings per mm), coupled to a nitrogen cooled front-illuminated charge-coupled device (CCD) detector. Acquisition and basic treatments of spectra (determination of peak positions and full width at half maximum (FWHM) of the Raman bands) have been performed using the LabSpec V5.25 (Jobin-Yvon, Horiba Group, Kyoto, Japan) software.

## Results and discussion

### X-ray powder diffraction analysis (XRPD)

**Fig. 1:**
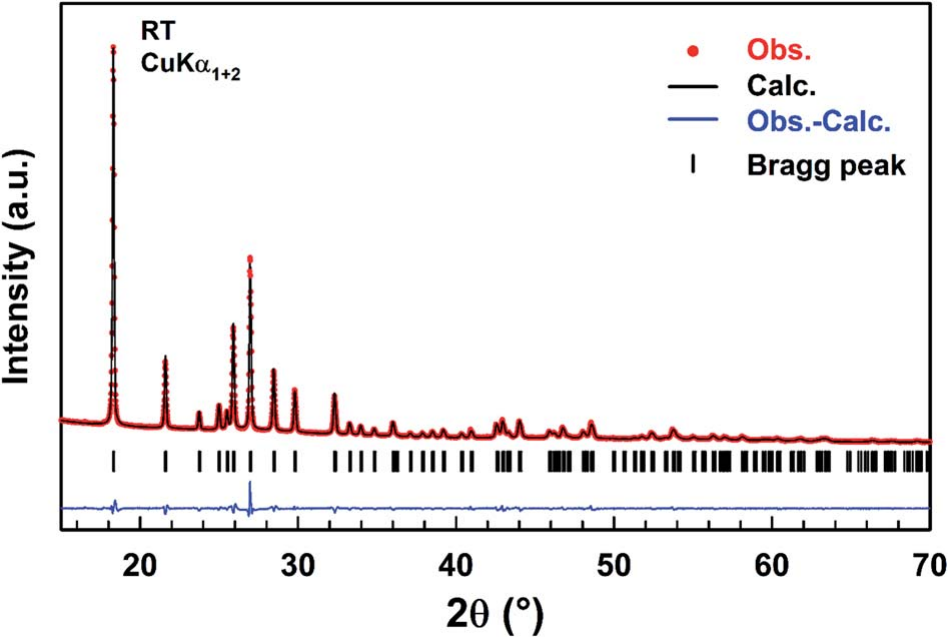
Observed (red dots), calculated (black line), and difference (blue line) diffraction patterns of [(C_2_H_5_)_4_N]FeCl_4_ compound. Vertical markers give Bragg peak positions.

A refinement of the XRPD pattern collected at room temperature was carried out by the Le Bail method using the cell parameters and the *P*6_3_*mc* (*n* = 186) space group reported in the literature as a starting point.^25^Fig. 1 shows the observed, calculated, and difference diffraction patterns for [(C_2_H_5_)_4_N]FeCl_4_. All Bragg peaks were successfully indexed and satisfactorily modelled, thus confirming the high purity of the sample. The conventional reliability factors of the refinement are *R*_p_ = 11.2%, *R*_ωp_ = 8.20%, *R*_exp_ = 3.08% and *χ*^2^ = 7.07. The hexagonal cell parameters, *a* = 8.2248(5) Å and *c* = 13.2159(8) Å, are in good agreement with those determined from XRD data collected on a single crystal of ([(C_2_H_5_)_4_N]FeCl_4_.^30^

### UV/vis spectroscopy

**Fig. 2:**
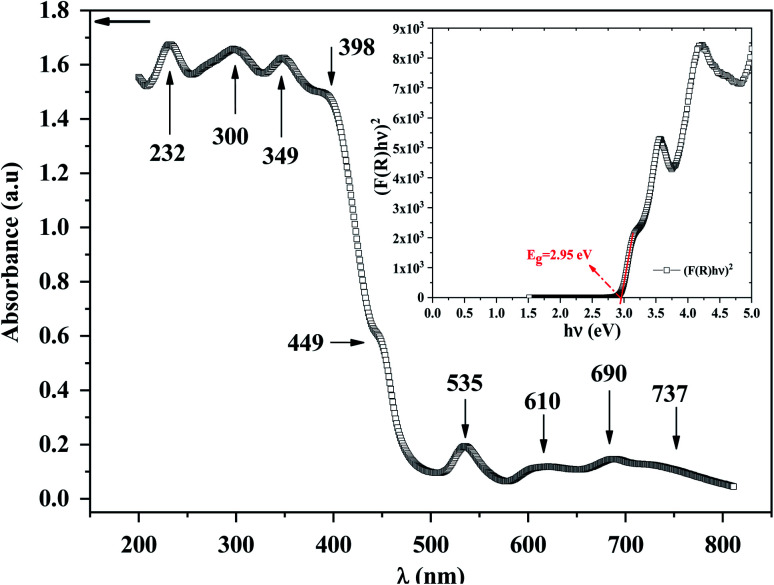
UV-vis absorption of [(C_2_H_5_)_4_N]FeCl_4_ compound. Insert: the Tauc plot.

To explore the semiconducting properties of [(C_2_H_5_)_4_N]FeCl_4_ compound, a solid-state UV-vis spectroscopy analysis was performed. The experimental absorption spectrum of the [(C_2_H_5_)_4_N]FeCl_4_ complex is plotted in Fig. 2. Strong absorption is observed in the spectral region roughly between 200 and 400 nm then it decreases sharply from 400 to 500 nm, thus indicating the presence of the band gap.^31^ The absorption bands are characteristic of the electronic transitions within the inorganic part ([FeCl_4_]^−^ anion), rather than the cation group, since the organic molecules are transparent in the visible spectral range.^32^ The five characteristic bands located at 232, 300, 349, 398 and 449 nm are commonly assigned to [FeCl_4_]^−^ anion.^33,34^ The other bands observed at 535, 610, 690 and at 737 nm, assigned to ^6^A_1_ → ^2^T_1_(a), ^4^E(b) (^6^A_1_ → ^4^T_2_(b)), ^6^A_1_ → ^4^A_2_, ^6^A_1_ → ^4^T_2_(a) and ^6^A_1_ → ^4^T_1_(a) transitions respectively, are characteristic of the [FeCl_4_]^−^ anion in a tetrahedral geometry.^35^

According to R. E. Marotti *et al.*^36^ and R. Henríquez *et al.*,^37^ the shoulders which appear in the reflectance spectrum *R* occur at wavelengths corresponding to the optical gap. The shoulders can be highlighted by calculating the first derivative (1/*R*)(d*R*/d*λ*). This magnitude variation as a function of the wavelength is presented in Fig. S1.† The presence of a strong peak centered at 421 nm allows to estimate the energy of the optical gap at 2.95 eV according to the relationship between *E*_g_ and *λ*:1
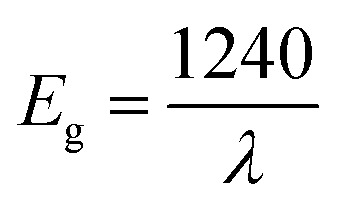


The gap energy *E*_g_ of materials is also linked to the absorption coefficient (*α*) by the following Tauc relation:^38^

2 *αhν* = *A*(*hν* − *E*_g_)^*n*^

where *A* is the absorption constant, *h* is Planck's constant, *ν* is the frequency of light and *n* has an index related to the of optical absorption process. *n* takes two values depending on the nature of the transition allowed. Theoretically *n* is equal to 1/2 for a direct transition and *n* = 2 in the case of an authorized indirect transition. Usually for powdered materials (supposedly opaque), the most appropriate method, to deduce the absorption coefficient, is that of Kubelka–Munk (K–M or *F*(*R*)) function:^39^3
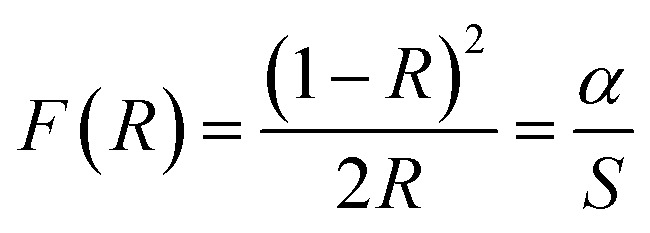
where *R* represents the reflectance and *S* is the thickness.

Using both the K–M function and the Tauc equation, we get:4
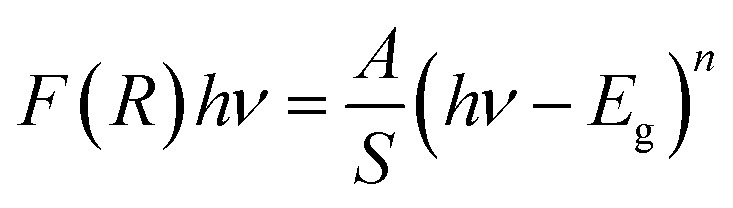


In inset Fig. 2, the *x*-intercept of the linear region of the curve (*F*(*R*)*hν*)^2^ occurs at 2.95 eV. This value is very close to the one obtained by Marotti method. Such energy gap value, significant light absorption in the visible range and indicates that of this semi-conducting [(C_2_H_5_)_4_N]FeCl_4_ compound is more appropriate for photocatalysis process using blue radiation. Inlike TiO_2_, which is widely used as heterogeneous catalysis with band gap energy of 3.2 eV (ref. 40) and requiring UV radiation, [(C_2_H_5_)_4_N]FeCl_4_ has a considerable advantage in the field of photocatalysis since visible light can be used. [(C_2_H_5_)_4_N]FeCl_4_ which is insoluble in toluene functions as a photocatalyst by dissociating chlorine atoms, which abstracts hydrogen from toluene, and photooxidation only occurs to the extent that benzyl alcohol and benzaldehyde.^20^ This low cost compound has a potential to lead the photooxidation of other hydrocarbons and therefore it can be an important material in the synthesis of pharmaceuticals (alcohols).

### Thermal analysis and temperature-controlled X-ray powder diffraction

According to Differential Scanning Calorimetry (DSC) data shown in Fig. S2,† it appears on the first heating/cooling run a large endothermic peak around 373 K which is no longer observed in the second run. Because chlorides are sensitive to moisture, water molecules can be easily adsorbed onto the surface of crystals. This large endothermic peak is thereby ascribed to a dehydration of [(C_2_H_5_)_4_N]FeCl_4_ sample. XRPD patterns collected at room temperature before and after a dehydration of the sample at 373 K (Fig. S3†) show that the crystal structure is preserved since no trace of secondary phase is detected. So, all DSC thermograms, Raman spectra and XRPD patterns analyzed hereafter were recorded after first dehydrating the sample at 373 K (*i.e.* during a second heating).

**Fig. 3:**
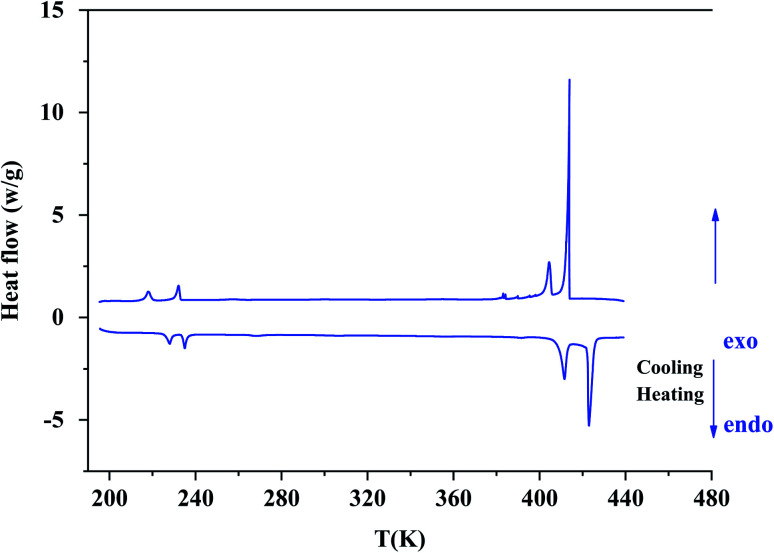
The second heating/cooling run for [(C_2_H_5_)_4_N][FeCl_4_] compound in the temperature range from 193 K to 443 K.

**Fig. 4:**
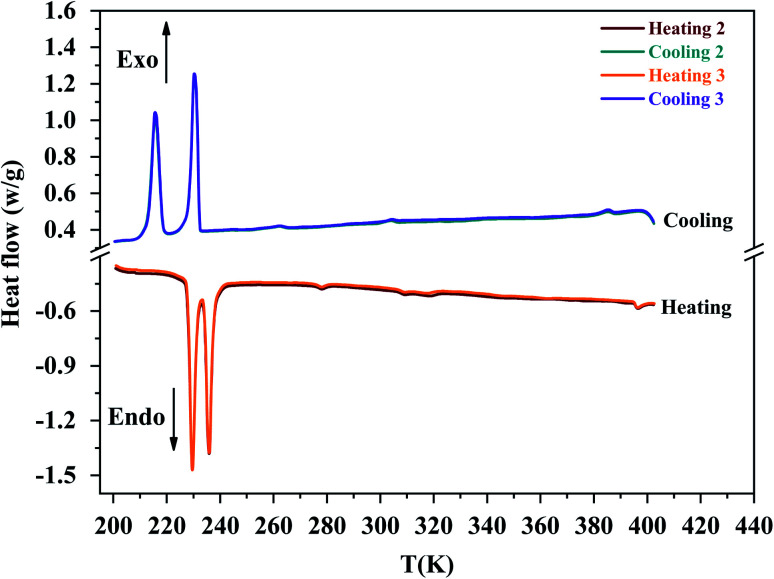
The DSC plot for [(C_2_H_5_)_4_N][FeCl_4_] compound in the temperature range from 193 K to 406 K.

On DSC thermograms displayed in Fig. 3, the sample exhibits upon the second heating run eight endothermic anomalies at 228, 235, 268, 306, 355, 392, 412 and 423 K. Only four of these anomalies were previously reported in literature (*i.e.* 228, 235, 412 and 423 K).^23,25–27^ The sharp and intense endothermic peak at 423 K is associated with a high entropy value (Δ*S* = 22.16 J K^−1^ mol^−1^) which is comparable to entropy values measured during solid-plastic transition of crystals such as [(CH_3_)_4_N]FeCl_4_ (Δ*S* = 13.5 J K^−1^ mol^−1^),^24^, [(CH_3_)_4_N]FeBrCl_3_ (Δ*S* = 15.6 J K^−1^ mol^−1^),^21^, [(C_2_H_5_)_4_N]BF_4_ (Δ*S* = 22.0 J K^−1^ mol^−1^)^41^ and (C_2_H_4_N_3_)(C_4_F_9_SO_3_) (Δ*S* = 26.0 J K^−1^ mol^−1^).^42^ The endothermic peak at *T*_*t*_ = 412 K, slightly less intense than the peak at 423 K, was only observed upon the second heating run, that is to say after dehydrating the sample. Upon the second cooling down, two exothermic peaks are observed at 414 and 404 K revealing that these two high-temperature phase transitions are reversible in nature. A second series of DSC analysis was carried out in the temperature range 193–406 K (below the solid-plastic transition). The corresponding DSC thermograms are displayed in Fig. 4. By discarding the first heating–cooling run used to dehydrate the sample (as previously mentioned), the same six peaks are still detected upon second heating run and the same number at the next cooling (Table 1). These six phase transitions are therefore reversible in nature. A total of eight phase transitions is evidenced by DSC analysis (Table 2) which implies the existence of nine crystal phases. The phase transition nature are identifying using the Boltzmann equation Δ*S* = *R* ln(*N*), where *R* is the gas constant and *N* represents ratio of the number of respective geometrically distinguishable orientations.^43^ An order–disorder transition can be interpreted if the *N* value is greater than two (*N* > 2).

**Table 1:** Phase transition temperatures determined by DSC in the temperature range 193–406 K. The sample was first heated up to 373 K (run 1) before recording successively run 2 and 3

Run 2		Run 3	
Heating (K)	Cooling (K)	Heating (K)	Cooling (K)
230	216	230	216
236	230	236	230
278	262	278	262
309	289	309	288
318	304	317	304
396	385	396	385

**Table 2:** Heat changes and the nature of phase transitions^a^

Heating					Cooling		
*T* (K)	Δ*H* (J mol^−1^)	Δ*S* (J mol^−1^ K^−1^)	*N*	Transition nature	*T* (K)	Δ*H* (J mol^−1^)	Δ*S* (J mol^−1^ K^−1^)
**Fig. 2(a)** **[193 K–443 K] (Run 2)**							
228.02	1241.55	5.44	1.92	*	218	1250.68	5.74
235	1193.36	5.08	1.84	*	232.12	1408.49	6.07
268.36	366.26	1.36	1.18	*	257.6	199.77	0.77
306.42	189.69	0.62	1.08	*	300.68	119.10	0.40
355	107.33	0.30			355	221.01	0.62
391.6	177.24	0.45	1.06	*			
411.57	5302.84	12.88	4.70	+	404.49	3370.26	8.33
422.94	9370.72	22.16	14.37	+	413.88	12 925.25	31.23

**Fig. 2(b)** **[193 K–406 K] (Run 2)**							
229.59	1853.13	8.07	2.64	+	215.64	1950.20	9.04
235.88	1618.96	6.86	2.28	+	230.39	2050.75	8.89
278.04	56.60	0.20	1.02	*	262.17	81.80	0.31
309.22	22.93	0.07	1.00	*	304.1	48.71	0.16
318.45	35.40	0.11	1.01	*			
396.35	96.68	0.18	1.02	*	385.3	66.33	0.17

**Fig. 2(b)** **[193 K–406 K] (Run 3)**							
229.59	1839.45	8.01	2.62	+	215.64	1962.99	9.10
235.88	1679.95	7.12	2.36	+	230.39	2041.77	8.86
278.04	59.34	0.21	1.03	*	262.17	74.12	0.28
309.04	21.78	0.07	1.00	*	304.25	50.54	0.17
317.3	47.53	0.15	1.02	*			
396.35	76.82	0.19	1.02	*	385.3	55.41	0.14

a *: not purely order disorder transition, +: order disorder transition.

**Fig. 5:**
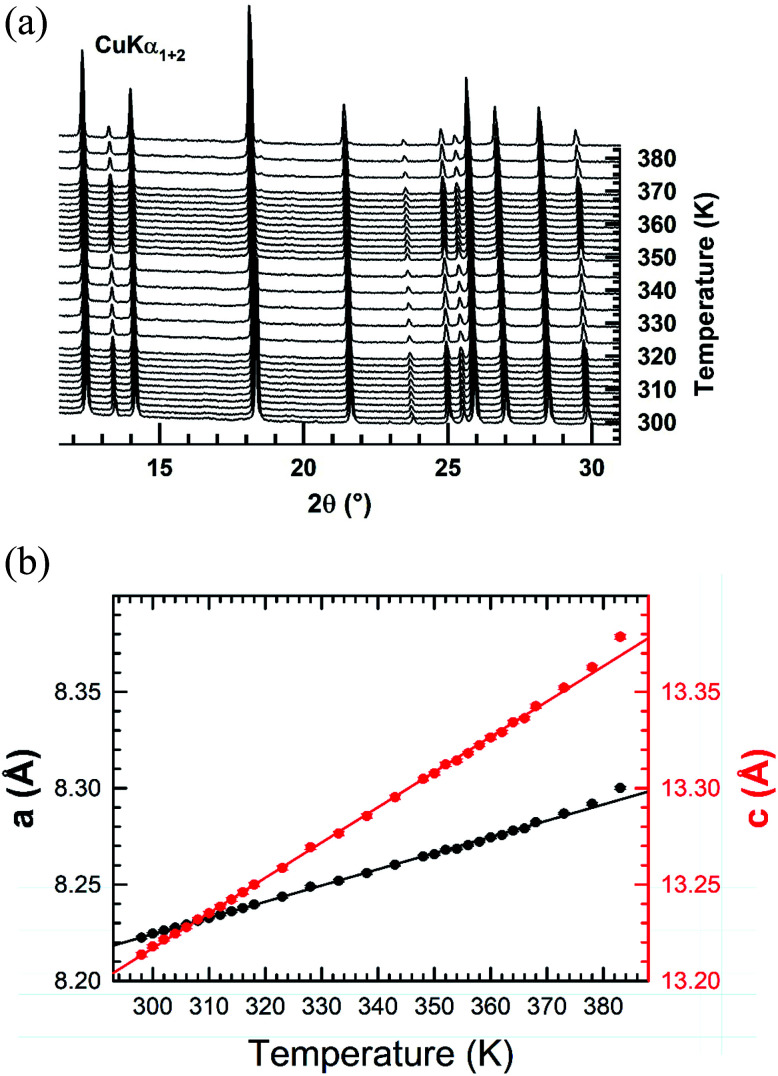
(a) XRPD patterns collected upon heating in air from RT to 383 K after *in situ* dehydrating the [(C_2_H_5_)_4_N]FeCl_4_ compound at 373 K. (b) Thermal evolutions of the hexagonal cell parameters *a* and *c* of the [(C_2_H_5_)_4_N]FeCl_4_ compound determined from the refinement of the XRPD patterns by the Le Bail method.

Temperature-controlled X-ray diffraction patterns were collected in the temperature range 298–383 K in order to detect any change in the crystal structure caused by the transitions detected below 383 K by DSC. No measurements were carried out above 383 K in order to avoid any reaction of the sample with the glass ceramic holder cup. The refinement of each XRPD pattern was carried out by the Le Bail method. Successively, the hexagonal cell parameters determined from the refinement of a lower temperature XRPD pattern was used as starting values for the refinement of the next higher temperature one. The thermal evolutions of the XRPD patterns together with those of the cell parameters *a* and *c* are displayed in Fig. 5. No change in the shape of peaks or appearance of new ones is noted in the collected XRPD patterns as function of temperature. This suggests that the transitions at 309, 318 are likely due to weak displacements/reorientations of the organic [(C_2_H_5_)_4_N]^+^ cation in the crystal structure of [(C_2_H_5_)_4_N]FeCl_4_. Both cell parameters exhibit a linear expansion with a slight departure from linearity above 379 K. The thermal expansion coefficients along the *a* and *c* crystallographic axes are 8.41 × 10^−4^ K^−1^ and 1.83 × 10^−3^ K^−1^ in the range 298–378 K, respectively. The thermal expansion along the [001] direction is much larger in magnitude than the one in the basal (*a*,*b*) plane.

### Temperature evolution of the Raman spectra

At room temperature, vibrational band assignments (Table 3) were performed based on previous reported data.^44–46^ The vibrational modes of [FeCl_4_]^−^ anion appear in the 70–400 cm^−1^ wavenumber range and the bands located above 400 cm^−1^ can be attributed to the vibrational modes of the cationic part ([(C_2_H_5_)_4_N]^+^).

**Table 3:** Observed Raman frequencies in (cm^−1^) of [(C_2_H_5_)_4_N]FeCl_4_ compound^a^

Raman position (cm^−1^)	Vibration assignment	Intensity
**[FeCl**_**4**_ **]** ^**−**^		
120	δ_s_(FeCl_4_)	Strong
140	δ_as_(FeCl_4_)	Medium
331	ν_s_(FeCl_4_)	Very strong
380	ν_as_(FeCl_4_)	Weak
392	ν_as_(FeCl_4_)	Medium

**[(C**_**2**_ **H**_**5**_ **)**_**4**_ **N]** ^**+**^		
470	δ(C–C–N)	Weak
517	—	Very weak
552	δ_s_(C–C–N)	Very weak
664	ν_s_(NC_4_)	Medium
792	ν_a_(NC_4_)	Very weak
892	ν(C–C)	Medium
1008	ν_s_(C–C–N)	Weak
1032	ν_s_(C–C–N)	Weak
1069	ν_a_(C–C–N)	Medium
1079	ν_a_(C–C–N)	Weak
1121	β(C–C–H)	Weak
1148	β(C–C–H)	Very weak
1187	ρ(CH_3_)	Very weak
1303	τ(CH_2_) + ω(CH_2_)	Weak
1354	δ_s_(CH_3_)	Weak
1400	δ_a_(CH_3_)	Weak
1441	δ(CH_2_)	Weak
1461	δ(CH_2_)	Medium
2890	—	Weak
2939	—	Very weak
2951	ν_s_(CH_2_)	Medium
2980	ν_a_(CH_2_)	Medium
2988	ν_s_(CH_3_)	Medium
2998	—	Medium
3020	ν_a_(CH_3_)	Weak
3112	—	Very weak
3129	—	Weak

a ν_s_: symmetric stretching; ν_s_: asymmetric stretching; δ_s_: symmetric bending; δ_s_: asymmetric bending; t: twisting; ρ_r_: rocking, τ: torsion, β: bending.

Based on DSC results and the XRPD patterns, the crystals are heated beforehand to 373 K then cooled down to room temperature before studying the phase transitions by Raman spectroscopy. No change was observed in the Raman spectra after sample dehydration (Fig. S5†).

**Fig. 6:**
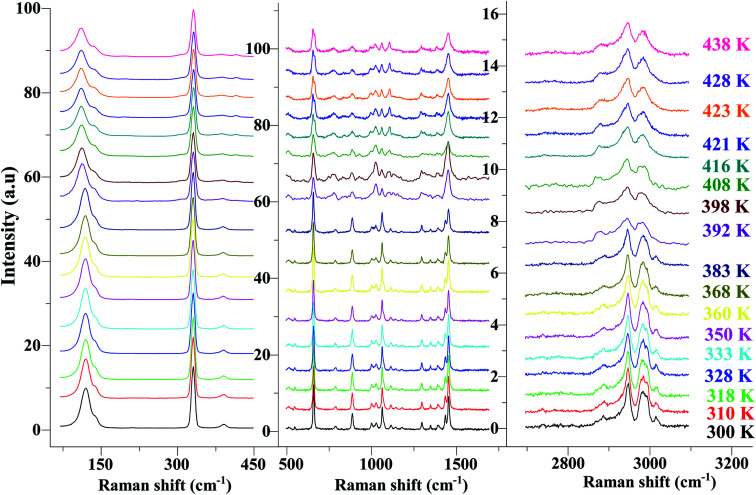
Temperature evolution of the Raman spectra for selected spectral ranges [70–450 cm^−1^], [500–1700 cm^−1^] and [2700–3200 cm^−1^].

**Fig. 7:**
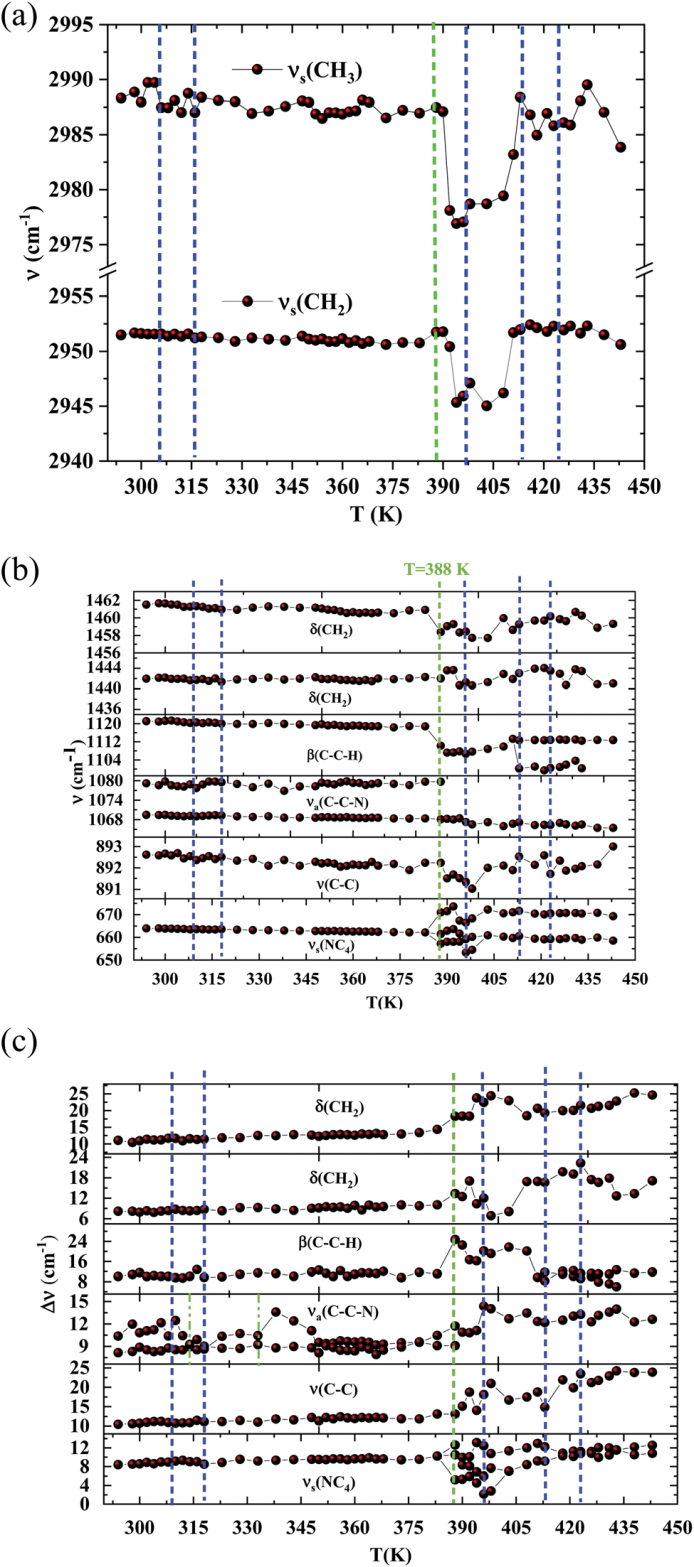
(a) Raman wavenumbers of the CH_2_ and CH_3_ asymmetric stretching *versus* temperature. (DSC transitions in blue vertical lines. The green line shows a Raman transition which is not detected by the DSC). (b) Raman wavenumbers of the organic part *versus* temperature. (DSC transitions in blue vertical lines. The green line shows a Raman transition which is not detected by the DSC). (c) Half widths of the Raman bands associated with [(C_2_H_5_)_4_N]^+^*versus* temperature. (DSC transitions in blue vertical lines. The green line shows a Raman transition which is not detected by the DSC).

**Fig. 8:**
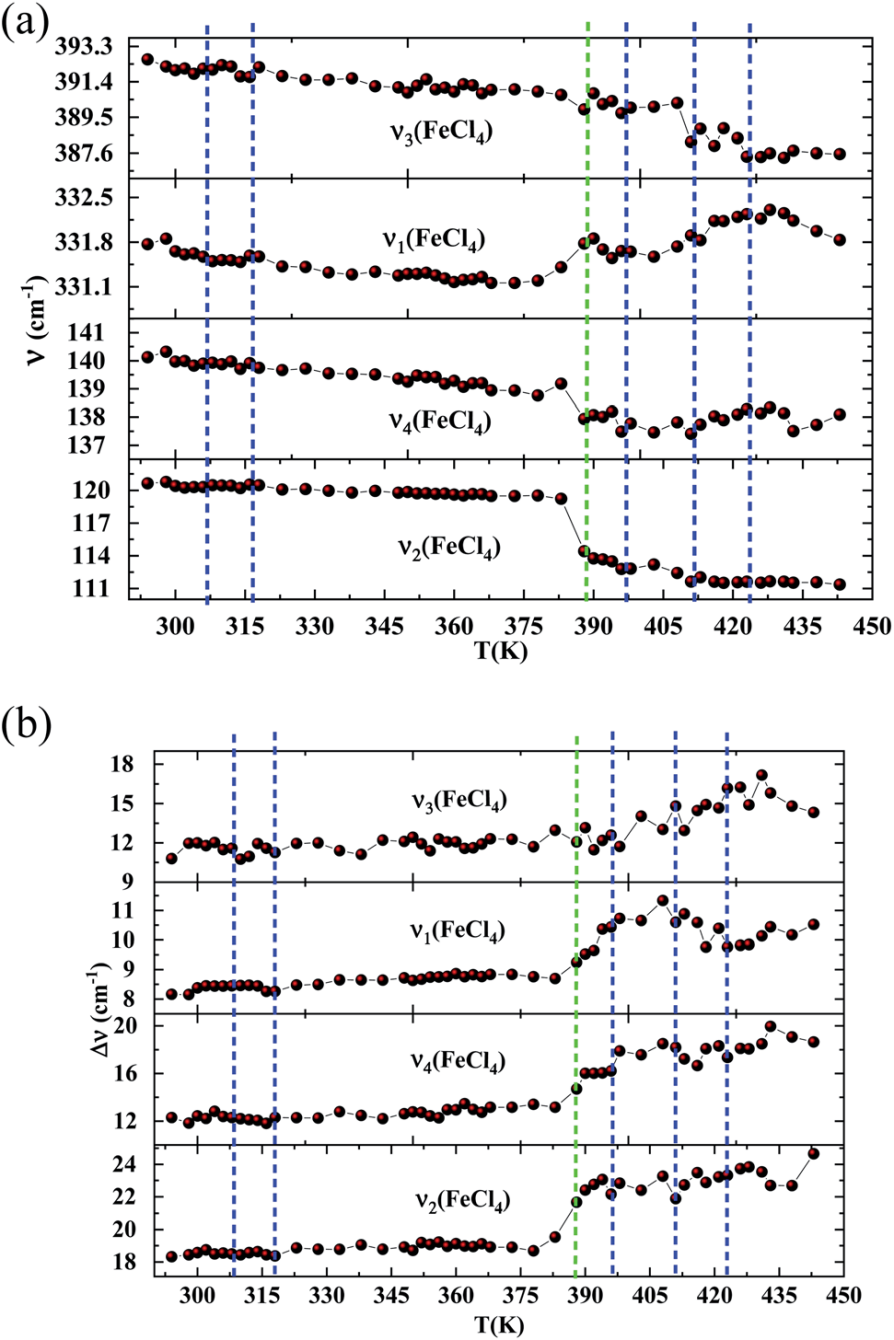
(a) Raman wavenumbers of the inorganic part *versus* temperature. (DSC transitions in blue vertical lines. The green line shows a Raman transition which is not detected by the DSC). (b) Half widths of the Raman bands associated with [FeCl_4_]^−^*versus* temperature. (DSC transitions in blue vertical lines. The green line shows a Raman transition which is not detected by the DSC).

In order to highlight the reorientations of organic cations involved during the different phase transitions, a large number of spectra (50 spectra) were recorded in the temperature range 294–443 K. For more clarity, Fig. 6 presents only selected spectra recorded on the three wavenumber ranges ([2700–3100 cm^−1^], [500–1700 cm^−1^] and [70–450 cm^−1^]). The temperature dependence of the peak positions and the full width at half maximum of the main Raman bands are illustrated in Fig. 7 and 8.

The bands observed between 2800 and 3200 cm^−1^ are commonly assigned to the symmetric and antisymmetric vibration of CH_2_ and CH_3_ groups. The two strongest bands, located at 2951 and 2988 cm^−1^ show changes in position with temperature since a shift of about 5 cm^−1^ has been observed between 388 and 413 K (Fig. 7(a)).

The Raman bands assigned either to the stretching or the deformation vibrations of the alkyl chains of the tetraethyl-ammonium cation [(C_2_H_5_)_4_N]^+^ are also sensitive indicators to phase transitions. The band at 665 cm^−1^, assigned to ν_s_(NC_4_), is first divided into three bands at 388 K and merged into two lines at 396 K. The vibrational modes at 892 cm^−1^ (ν(C–C)), 1441 and 1461 cm^−1^ (δ(CH_2_)) show, respectively, a jump towards the high and low frequencies at the transition temperatures, associated with a variation of the full width at half maximum (Table 4). Another important spectral modification was observed at 388 K in the wavenumber range 1050–1100 cm^−1^ where two vibrational bands merged into a single one. The band at 1121 cm^−1^ assigned to β(C–C–H) show a shift of 9 cm^−1^ at 383 K before splitting into two bands around 413 K and a broadening of about 12 cm^−1^ between these two temperatures. Such results suggest a reorientation of the organic [(C_2_H_5_)_4_N]^+^ cation at 388 K and a more pronounced disordering of the alkyl chains above 416 K as confirmed by the broadening of the bandwidth of the C–C and C–C–N stretching modes.

**Table 4:** The wavenumbers and half widths variation of the Raman bands around the transition temperatures^a^

*T*_*t*_ (K)		*T*_1_ = 313		*T*_2_ = 388		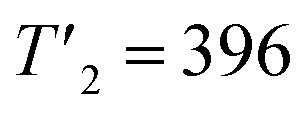		*T*_3_ = 413		*T*_4_ = 423	
Wavenumber (cm^−1^)	Assignment	*ν* (cm^−1^)	Δ*ν* (cm^−1^)	*ν* (cm^−1^)	Δ*ν* (cm^−1^)	*ν* (cm^−1^)	Δ*ν* (cm^−1^)	*ν* (cm^−1^)	Δ*ν* (cm^−1^)	*ν* (cm^−1^)	Δ*ν* (cm^−1^)
**Inorganic part**											
120	ν_2_(FeCl_4_)			−5	+3						
140	ν_4_(FeCl_4_)			−2	+2						
331	ν_1_(FeCl_4_)			+0.7	+0.5						−0.7
392	ν_3_(FeCl_4_)			−1	−0.8		+2	−2		−1	

**Organic part**											
664	ν_s_(NC_4_)			+9	+3	−1	−1	−1	−1		
				−1		−2	+1				
				−5	−5	−5	+5	−1	+1		+1
892	ν(C–C)			−1	+2	+1	+4	+0.7	+7	−1	+3
								−0.5		+0.5	
1069	ν_a_(C–C–N)										
1079	ν_a_(C–C–N)		−1	−11	+1	−1	+3	−1		+1	−1
1121	β(C–C–H)		+2	−9	+14	+2	+4	−1	+2		−1
								−13	−2		−1
1441	δ(CH_3_)	+1		+2	+3	−2	−5		+3		−5
1461	δ(CH_2_)			−3	+4	−1	+6		−2		−2
2951	ν_s_(CH_2_)			−5				+5			
2988	ν_s_(CH_3_)	−2		−5				+5			

a *T*_*t*_: transition temperature; −: decreases; +: increases.

The vibration modes of [FeCl_4_]^−^ anions are other indicators sensitive to phase transitions. For instance, at 383 K the bands at 120 and 140 cm^−1^, attributed respectively to ν_2_(Fe–Cl and ν_4_(Fe–Cl), are shifted toward lower frequencies by 5 and 2 cm^−1^ (associated with a broadening of 3 and 2 cm^−1^), respectively. The band at 392 cm^−1^, assigned to ν_3_(Fe–Cl) shows a broadening of 2 cm^−1^ after the phase transition at 396 K and a shift of 2 cm^−1^ to lower wavenumber after the phase transition at 416 K. According to reported results on [(CH_3_)_4_N]FeCl_4_ compound,^24^ the series of modifications observed for [(C_2_H_5_)_4_N]FeCl_4_ around 383 K can be explained by a thermal activation of the rotation of [FeCl_4_]^−^ anions. This may explain the slight deviation from linearity above 379 K for the two crystallographic parameters *a* and *b*.

From all these results, we demonstrated that the most significant vibrational modifications are observed by Raman spectroscopy between 383 and 416 K. The Raman shifts and the broadening of the vibrational modes are more pronounced in this temperature range due to the interactions between the [(C_2_H_5_)_4_N]^+^ cations and the internal vibrations of the [FeCl_4_]^−^ anion. Above 416 K, our vibrational analysis confirms a disordered molecular structure of the hydrocarbon chains, characteristic of a plastic crystalline structure.

**Fig. 9:**
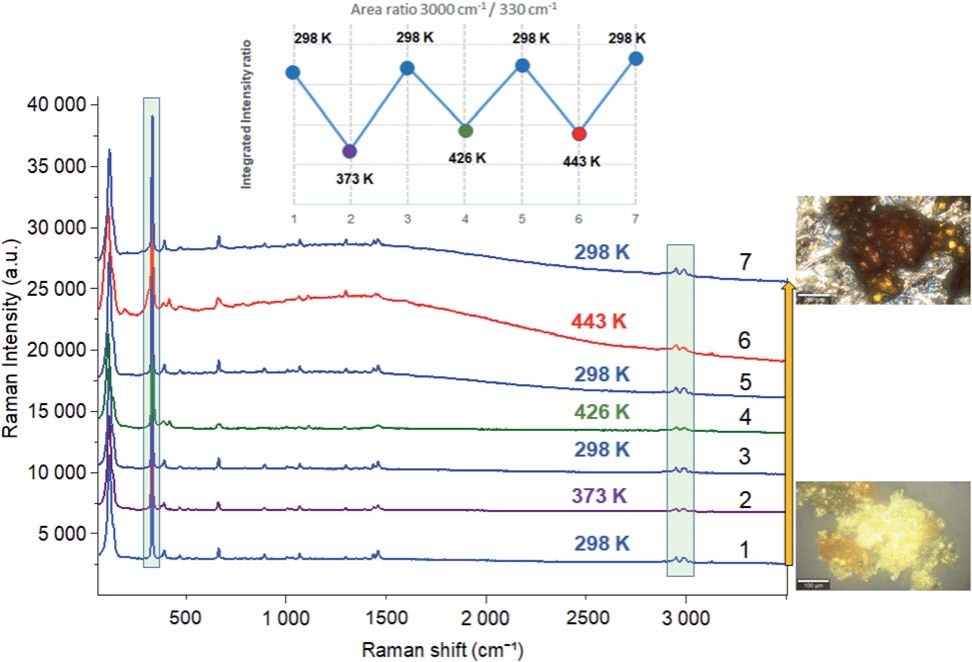
Raman spectra before and after heating to 373, 426 and 443 K.

In order to study the reversibility of the different phase transitions, Raman spectra were recorded before and after heating to 373, 426 and 443 K. The increase in baseline intensity observed at 443 K (Fig. 9 and S4†) may be associated with a change in color of the material. Let us notice this increase was not observed after heating to 373 or 426 K and then cooling down to 298 K. What is certainly the most interesting result is that the different temperature cycles do not change the position of the vibration bands, nor the intensity ratio of the vibrational bands characteristic of organic (at 3000 cm^−1^) and inorganic (330 cm^−1^) components (inset of Fig. 9) once the sample is returned to 298 K.

The solid–solid phase transition are likely to displacements/reorientations of the organic [(C_2_H_5_)_4_N]^+^ cation in the crystal structure of [(C_2_H_5_)_4_N]FeCl_4_. These molecular displacements are coherent with the electrical changes reported in our previous study.^25^ These switchable physical and/or chemical properties between two different states in response to temperature fluctuation could have potential applications in switches, sensors, memory devices, signal processing and so on.^47,48^

However, these switchable phase suffer small latent enthalpy changes limiting their applications as thermal energy storage material. In comparison, the plastic phase is accompanied by a much larger enthalpy change because the plastic phase transition often involves reorientational order–disorder and molecular displacements of the organic and inorganic components. It was shown that the hydrogen interactions between [FeCl_4_]^−^ anions and [(C_2_H_5_)_4_N]^+^ cations induce a locally disordered molecular structure after 423 K characteristic of the plastic state. Such a result suggests that the [(C_2_H_5_)_4_NFeCl_4_] as a plastic crystal was superior than other usual molecular phase transition materials as thermal energy storage materials. Therefore, combining the new function thermal energy storage with the multiple switching and photo-calytic application in [(C_2_H_5_)_4_NFeCl_4_] plastic crystal is a novel approach to develop multifunctional molecular materials.

## Conclusions

In summary, the [(C_2_H_5_)_4_N]FeCl_4_ compound was successfully prepared by slow evaporation solution growth method at room temperature. The significant light absorption in the visible range of this semi-conducting compound and a low gap energy of the order of 2.95 eV makes this material interesting in photocalytic domain with sunlight only.

We report a plastic crystal which presents solid–solid transitions and a plastic transition at 120 K above the ambient temperature. The suitable phase transition temperature (423 K), large thermal energy storage capacity (9.37 kJ mol^−1^ for the latent heat storage, and totally ∼18 kJ mol^−1^ from 293 to 443 K), the high thermal stability, good thermal conductivity,^25^ easy preparation and processability suggest that the [(C_2_H_4_)_4_N]FeCl_4_ is an excellent candidate as thermal energy storage materials. The multi-switchable phases have been reported for the first time in the [(C_2_H_4_)_4_N]FeCl_4_ single-phase plastic crystal. These switchable physical states could have potential applications in switches, sensors, memory devices, and so on. This work not only exhibits the advantage of the molecular plastic phase transition materials as thermal energy storage materials but also provides a new design principle for multifunctional molecular material. Nevertheless, the magnetic and optical properties depending as a function of temperature in [(C_2_H_5_)_4_N]FeCl_4_ are perspectives in order to complete this work.

## Conflicts of interest

All authors have no conflicts of interest.

## Supplementary Material

RA-011-D1RA02475B-s001
