# Supplementary material for: Organic–inorganic interactions revealed by Raman spectroscopy during reversible phase transitions in semiconducting [(C2H5)4N]FeCl4
Source: RSC Adv. 2021 May 24;11(30):18651–60. doi: 10.1039/d1ra02475b (PMC9033484; doi:10.1039/d1ra02475b)
Supplement: RA-011-D1RA02475B-s001 [file RA-011-D1RA02475B-s001.pdf]

Electronic Supplementary Material (ESI) for RSC Advances.
This journal is © The Royal Society of Chemistry 2021

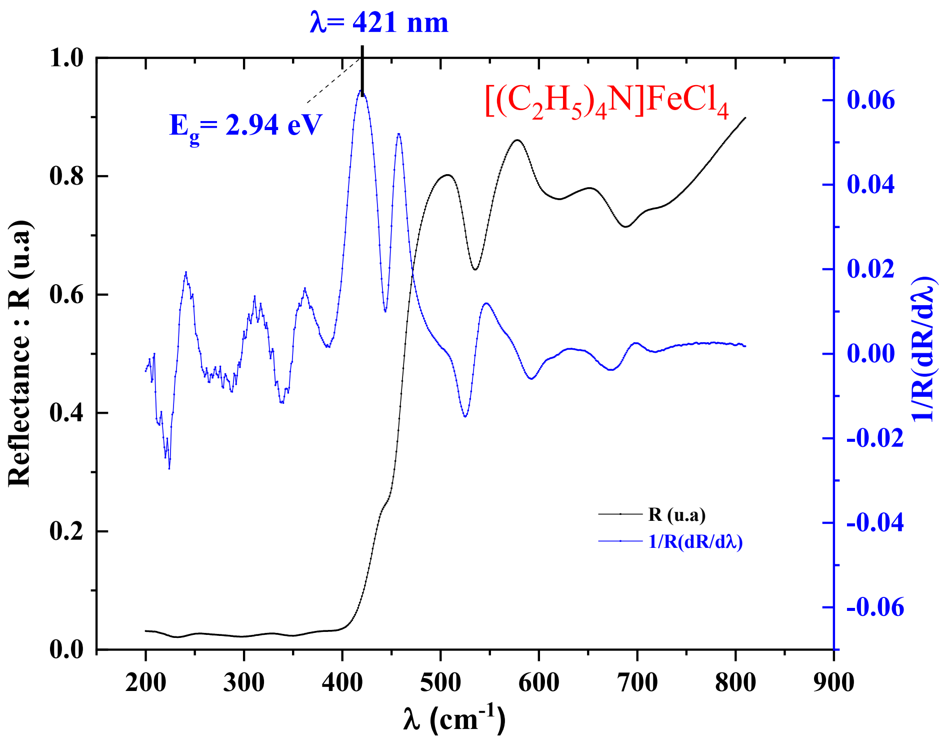

Fig. S1: $1/R(dR/d\lambda)$ versus λ of $[(C_2H_5)_4N]FeCl_4$ powder.

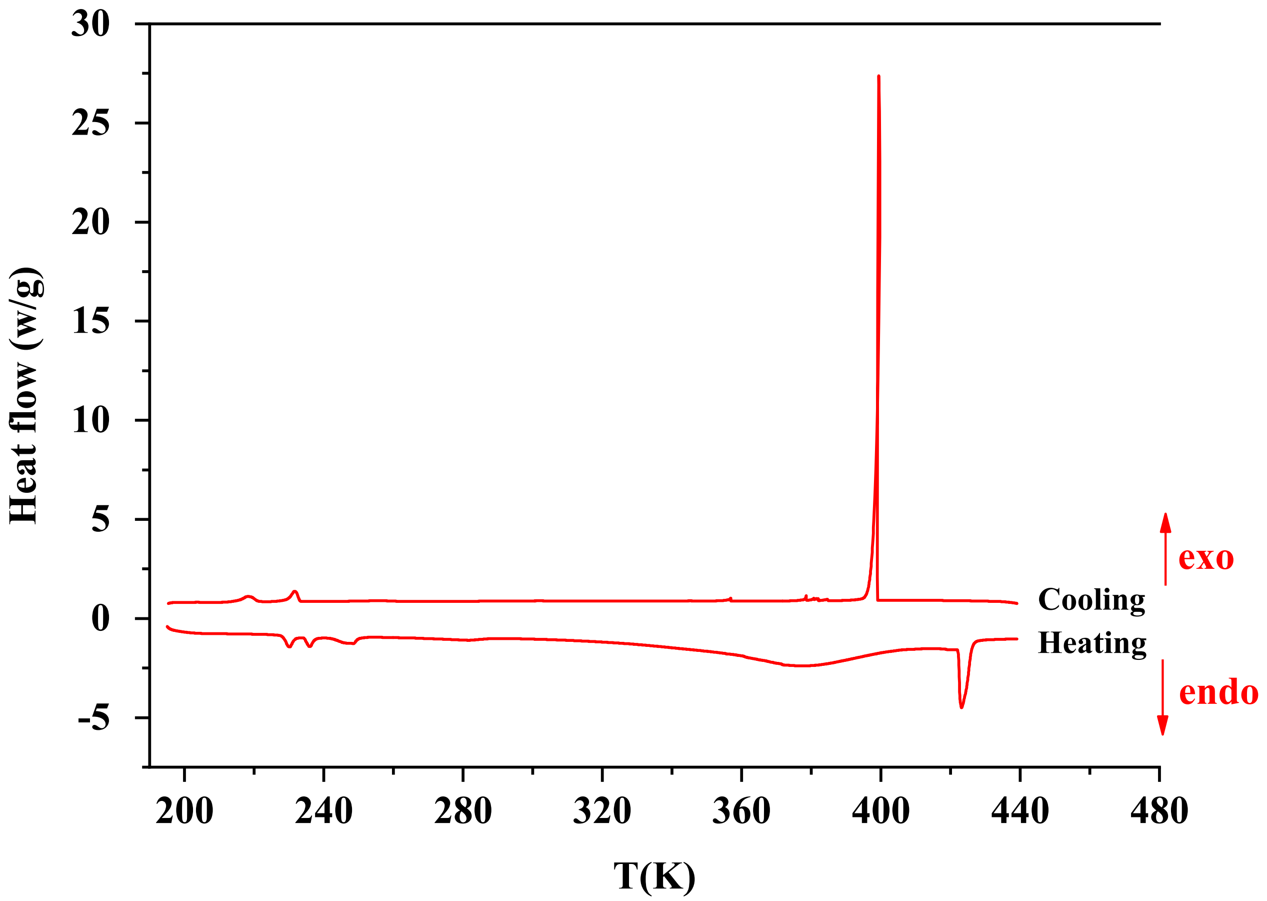

Fig. S2: First heating/cooling run between 193 and 443 K of the $[(C_2H_5)_4N]FeCl_4$ compound.

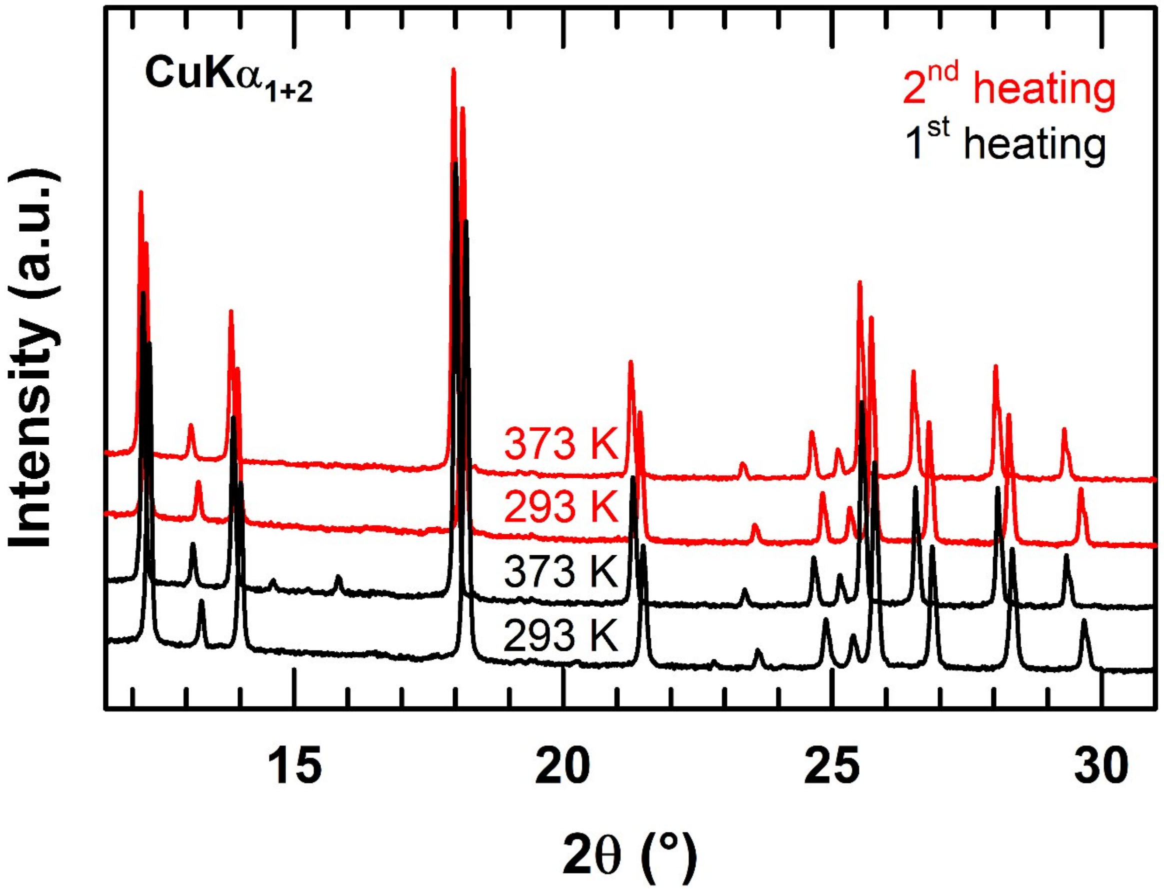

Fig. S3: X-ray powder diffraction at 293 and 373 K during two heating and cooling runs.

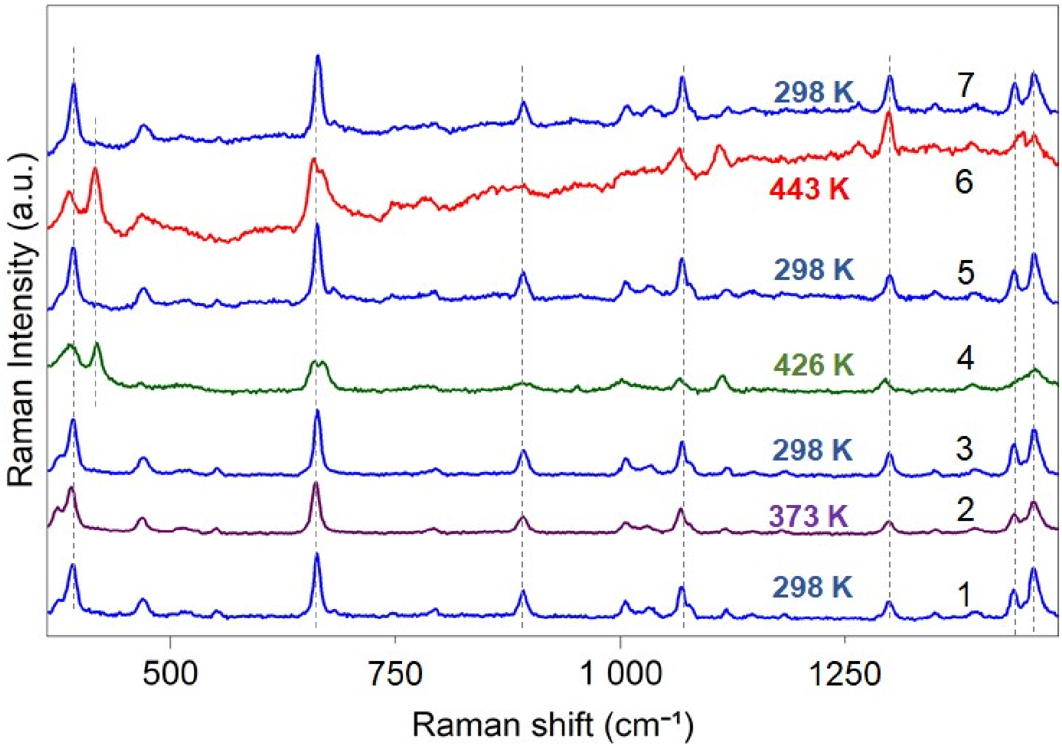

Fig. S5: Raman spectra before and after heating to 373, 426 and 443 K of $[(C_2H_5)_4N]FeCl_4$ compound.
